# Novel Application of Light-Emitting Diode Therapy in the Treatment of Eyebrow Loss in Frontal Fibrosing Alopecia

**DOI:** 10.3390/s21175981

**Published:** 2021-09-06

**Authors:** Agnieszka Gerkowicz, Joanna Bartosińska, Dorota Raczkiewicz, Mirosław Kwaśny, Dorota Krasowska

**Affiliations:** 1 Department of Dermatology, Venereology and Pediatric Dermatology, Medical University of Lublin, Staszica 11 St., 20-081 Lublin, Poland; dorota.krasowska@umlub.pl; 2 Department of Cosmetology and Aesthetic Medicine, Medical University of Lublin, Chodźki 1 St., 20-093 Lublin, Poland; joanna.bartosinska@umlub.pl; 3 Department of Medical Statistics, School of Public Health, Center of Postgraduate Medical Education, Kleczewska 61/63 St., 01-826 Warsaw, Poland; dorota.raczkiewicz@cmkp.edu.pl; 4 Institute of Optoelectronics, The Military University of Technology, Kaliskiego 2 St., 01-476 Warsaw, Poland; miroslaw.kwasny@wat.edu.pl

**Keywords:** frontal fibrosing alopecia, photobiomodulation, light-emitting diodes

## Abstract

Background: Eyebrow loss in the course of frontal fibrosing alopecia (FFA) is becoming a growing issue among older females. It has a considerable negative impact on patients’ quality of life. Since there is no standardized treatment, photobiomodulation with light-emitting diodes (LEDs) could be an option. Here we assess, for the first time, the efficacy of LED therapy in the treatment of eyebrow loss in females with FFA. Methods: 16 female patients with FFA aged 60–74 years were enrolled in the study. LED therapy was performed once a week for a 10-week session. The LEDs’ effectiveness was assessed at the baseline, after 10 irradiations, and 6 months after the end of treatment during a follow-up visit. Results: The therapy was well tolerated. After 10 irradiations, the total eyebrow hair count increased significantly, as did the number of thick hairs and mid-thick hairs (*p* = 0.002, *p* = 0.002, and *p* = 0.044, respectively). During the follow-up visit, the total number of eyebrow hairs remained significantly higher than before treatment (*p* = 0.002). Conclusion: The study revealed that LED therapy seems to be a novel and promising therapeutic option for eyebrow loss in patients with FFA. It is safe and well tolerated and leads to clinically and cosmetically acceptable improvement.

## 1. Introduction

The aging population is becoming an important public health issue worldwide. The percentage of people who are older has been increasing in the last 10 years and will continue to rise as a result of improved life expectancies [1]. Among the many conditions associated with aging, hair loss is becoming a growing problem. Balding may result not only from the physiological aging of hair follicles but also from various diseases or medications [2]. Older patients are more likely to worry about hair loss; however, this concern has often been overlooked both clinically and in the scientific community [2]. Thus far, there are only a few studies dealing with hair loss in older patients [2,3,4].

Frontal fibrosing alopecia (FFA) is a primary cicatricial alopecia characterized by progressive loss of the frontotemporal hairline and partial or total loss of the eyebrows. It is usually diagnosed in postmenopausal women with a mean age of 60 years, though it may also affect men and premenopausal women [5]. Recently, a rapid increase in FFA incidence has been observed worldwide [6,7]. Eyebrow loss has been found in the majority of patients, with some studies reporting rates of up to 94.6% [6]. This clinical manifestation has a negative impact on the patient’s quality of life [8]. Currently, there is no standardized treatment for eyebrow loss experienced in the course of FFA [9]. Given the need for novel therapies, photobiomodulation with the use of light-emitting diodes (LEDs) could be an option.

Photobiomodulation, otherwise known as low-level light therapy (LLLT), consists of the application of non-ionizing forms of light sources, including lasers or light-emitting diodes (LEDs), in the red or infrared spectrum with a relatively low power density <100 mW/cm^2^ and low fluencies in the range of 0.04–50 J/cm^2^ to alter biological activity in cells [10,11,12]. Possible mechanisms responsible for cellular photobiomodulation include increased adenosine triphosphate (ATP) production, the modulation of oxidative stress, and the induction of transcription factors [12,13]. Photobiomodulation has been demonstrated to be a promising therapeutic option for the treatment of Alzheimer’s dementia and Parkinson’s disease [14] as well as a way to improve the healing of ulcers among older patients [15]. Such a therapy is usually well tolerated, since it is a non-pharmacological treatment option and does not induce severe adverse effects [11,12,13,14,15,16]. Among a broad range of dermatological indications, LEDs have been used to treat hair loss [11,12]. Recently several studies have reported the effectiveness of LED therapy on scarring alopecia [17,18,19]. In our previous pilot study, we reported the favorable effect of LED therapy on the scalp hair of patients with FFA. That study demonstrated that LED irradiation was safe and well-tolerated and improved the outcome of FFA [17]. To the best of our knowledge, there are currently no studies evaluating LED therapy as a treatment for eyebrow loss in scarring alopecia. Given the promising results of a previous study [17], it was suggested that LED therapy could be beneficial in stimulating eyebrow regrowth in FFA.

The purpose of this study was to assess the effectiveness of LED irradiation in the treatment of eyebrow involvement in females with FFA.

## 2. Materials and Methods

### 2.1. Study Group

This study included 16 female patients with FFA aged 60–74 years, with an average age of 65 years. In all patients, the diagnosis of FFA was confirmed based on clinical presentation, the histopathological results of a scalp biopsy, and a trichoscopy of the scalp and eyebrow. To confirm eyebrow involvement, the presence of features typical for FFA—yellow/reddish/greyish dots, dystrophic hairs, whitish areas with the absence of a follicular opening, and eyebrow regrowth in distinct directions—was checked according to the criteria proposed by Waśkiel-Burnat et al. [20]. Trichoscopic examination was performed according to a standard procedure using the FotoFinder Dermoscope (FotoFinder System GmbH, Bad Birnbach, Germany).

The disease activity of FFA was assessed using the Frontal Fibrosing Alopecia Severity Score (FFASS) based on the protocol proposed by Saceda-Corralo et al., with a score of 0–25 [21].

The inclusion criteria were an age of 60 years or older, a disease duration of at least 1 year, no previous systemic treatment for FFA, no previous topical treatment for the eyebrow, and stable topical treatment of the scalp for six months. During the study, patients could not use any topical treatments for their eyebrows or begin any systemic treatment for FFA. Previously started topical treatments for the scalp were not modified and patients continued them.

The exclusion criteria included an age <60 years; epilepsy; non-scarring alopecia, including alopecia areata and trichotillomania; scarring alopecia other than FFA; previous systemic treatment for FFA or topical treatment of the eyebrow; beginning any new treatment for FFA during the study; and cosmetic procedures including eyebrow epilation.

The study was performed in accordance with the Declaration of Helsinki. All of the patients became acquainted with the objective of the study, and written informed consent was obtained from all of them before enrolment in the study. The study was approved by the Local Ethics Committee (decision no. KE-0254/167/2017).

### 2.2. Irradiation of the Eyebrows with LEDs

For all patients, the irradiations were performed using a source of light based on the LED matrix illuminator Red Beam pro+, Model APRO (MEDlight GmbH, Herford, Germany). This lamp contains three movable units with 78 high-powered red-light-emitting diodes of the latest generation, so-called superluminescent diodes (sLED), which provide red light operating at 630 ± 5 nm with a maximum power density of 100–120 mW/cm^2^. The therapy area corresponds to a minimum of 20 cm^2^. The dose per session was 37 J/cm^2^, and the light power density was 68 mW/cm^2^. The distance from the eyebrow was 15 cm. Each session lasted 9 min and 4 s.

The lamp was set to irradiate both eyebrows and the frontal hairline. Other parts of the face were not irradiated. During treatment, patients’ eyes were protected by goggles.

Treatment sessions were performed once a week for a period of 10 weeks from October to May. The therapy was not performed during the summer.

### 2.3. Assessment of the LEDs’ Efficacy

To determine the efficacy of LED irradiation on eyebrow growth impacted by FFA, trichoscopic examination was performed. During the trichoscopy both the left and right eyebrows were examined in 3 or 4 fields, depending on the length of the patient’s eyebrow area at 20× magnification (each field corresponds to 1 cm^2^). Within each field, all of the eyebrow hairs were measured using the dermatoscope’s dedicated firmware, which made the assessment of hair thickness possible and provided repeatable measurements.

After the trichoscopy, all the pictures with marked thickness of each eyebrow hair were downloaded from the dermoscope for counting. The eyebrow hairs were divided into 3 groups depending on their thickness: thick hairs measuring >0.05 mm, mid-thick hairs measuring 0.03–0.05 mm, and thin hairs measuring <0.03 mm. After adding the results of the thick, mid-thick, and thin eyebrow hair counts, the total eyebrow hair count was obtained. Counting was performed by 2 different investigators who did not perform the trichoscopy, and the results were compared. If the results were equal, then they were accepted; if there were differences, then the counting was repeated.

The measurement of eyebrow hair counts was performed three times: once before the treatment, once after 10 irradiations (1 week after the last irradiation), and once six months after the end of LED therapy during the post-treatment follow-up visit. Three groups were distinguished based on the baseline eyebrow hair count: total eyebrow hair loss (patients with no hairs in the eyebrows), almost total eyebrow hair loss (patients with 1–49 hairs in the eyebrows), and partial eyebrow hair loss (patients with >50 hairs in the eyebrows).

The primary endpoint of the study was the change in the total, thick, mid-thick, and thin eyebrow hair counts after 10 weeks. The secondary endpoint was the change in the total, thick, mid-thick, and thin eyebrow hair counts during the post-treatment follow-up visit.

### 2.4. Statistical Analysis

The data were statistically analyzed using the STATISTICA 13 software (StatSoft, Tulsa, OK, USA). The median and interquartile ranges (IQR) (lower quartile–upper quartile) were estimated for numerical variables, as were the absolute numbers (n) and percentages (%) of the occurrence of items for categorical variables. Wilcoxon’s signed-rank test was used to compare the number of hairs counted at the following endpoints: before and after the treatment, before the treatment and during the post-treatment follow-up visit, and after the treatment and during the post-treatment follow-up visit.

The significance level was assumed at 0.05.

## 3. Results

### 3.1. Characteristics of the Study Group

Detailed characteristics of the study group are presented in Table 1.

The study group included 16 females with FFA that had been ongoing for 3–10 years, 6 years on average. The severity of the disease, assessed according to FFASS, ranged between 4.7 and 19.3, 12 on average. Eyebrow loss was observed in all patients and had been ongoing for 3–10 years, 6 years on average.

All patients received 10 treatments; however, in three patients, there were some shifts in the therapy that lasted up to 2 weeks. Such gaps did not affect the treatment.

Among the trichoscopic features typical for FFA, the most common were greyish or yellow dots, which were observed in 14 (87.50%) patients, and eyebrow regrowth in a distinct direction, which was found in 11 (68.75%) patients.

In 12 (75.00%) patients, eyebrow involvement accompanied recession of the frontal hairline. In one (6.25%) patient, eyebrow loss was the first sign of FFA, while in three (18.75%) patients, it occurred later.

Before the treatment, one patient had total eyebrow hair loss (no hairs in the eyebrows), five (31.25%) patients had almost total eyebrow hair loss (1–49 hairs in the eyebrows), and 10 (62.50%) patients had partial eyebrow hair loss (>50 hairs in the eyebrows).

### 3.2. Comparison of Eyebrow Hair Counts before and after 10 Irradiations with LEDs

**Figure 1:**
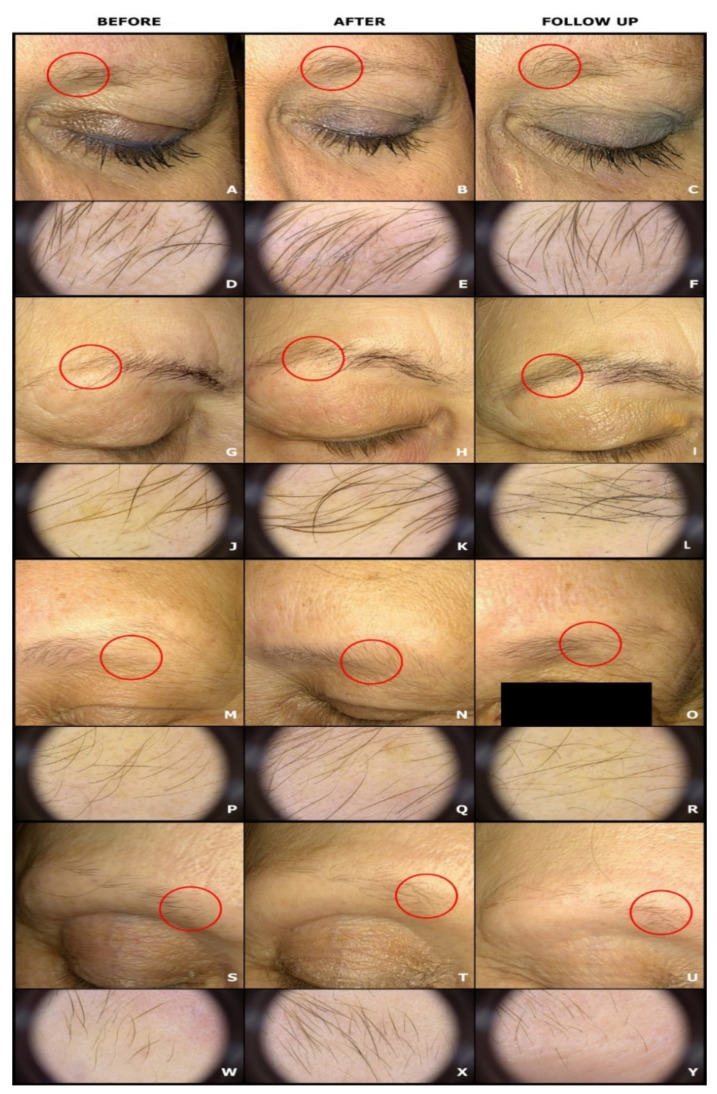
Comparison of clinical and trichoscopic images before (**A**,**D**,**G**,**J**,**M**,**P**,**S**,**W**), after (**B**,**E**,**H**,**K**,**N**,**Q**,**T**,**X**) 10 LED irradiations, and during the post-treatment follow-up visit (**C**,**F**,**I**,**L**,**O**,**R**,**U**,**Y**). The red circle indicates localization of compared trichoscopic images. 1E, 1K, 1Q, 1X: increased number of thick eyebrow hairs and increased total eyebrow hair count after 10 LED irradiations; 1F, 1L, 1R, 1Y: reduced number of thick hairs compared with the post-treatment images, and similar or increased total eyebrow hair count compared with the trichoscopic images at baseline.

Across the whole study group, total eyebrow hair counts increased significantly from 132 on average before LED therapy to 152 on average after the treatment (*p* = 0.002). Thick eyebrow hair counts also increased significantly from 80 on average before irradiations to 111 on average after the treatment (*p* = 0.002). Mid-thick eyebrow hair counts increased significantly as well from 28 on average before LED therapy to 34 on average after the treatment (*p* = 0.044). Thin eyebrow hair counts did not change significantly after the treatment (*p* = 0.999). The results are shown in Table 2 and Figure 1.

After 10 LED irradiations on the patient with total eyebrow hair loss, eight eyebrow hairs appeared. In two patients with almost total eyebrow hair loss, the total eyebrow hair count increased by 11 and 37, while in two patients it did not change and in one patient it decreased by nine. In 10 patients with partial eyebrow hair loss, the total eyebrow hair count increased by 9 to 106 eyebrow hairs.

### 3.3. Comparison of Eyebrow Hair Counts before the Treatment and during the Post-Treatment Follow-Up Visit

The total number of hairs within the eyebrow was significantly higher during the follow-up visit than before the treatment (176 vs. 132 on average), *p* = 0.002. Moreover, thickness-specific eyebrow hair counts increased significantly during the follow-up visit in comparison with before the treatment (*p* = 0.033 for thick hairs, *p* = 0.019 for mid-thick hairs, and *p* = 0.038 for thin hairs). The results are shown in Table 2 and Figure 1.

In the case of the patient who had total eyebrow hair loss before the treatment, eight eyebrow hairs, which appeared after the treatment, were still observed during the post-treatment follow-up visit. During the post-treatment follow-up visit, the total eyebrow hair counts were higher than the pre-therapy counts by 10 and 31 for two patients who had almost total eyebrow hair loss. For two patients, the counts did not change, and for one patient, the count during the post-treatment follow-up visit was lower by 19. For all patients with partial eyebrow hair loss, the total eyebrow hair counts taken during the post-treatment follow-up visit were higher by 7 to 155 eyebrow hairs.

### 3.4. Comparison of Eyebrow Hair Counts after 10 Irradiations with LEDs and during the Post-Treatment Follow-Up Visit

The total eyebrow hair count as well as the mid-thick hair count did not change significantly after the treatment and during the follow-up visit (*p* = 0.623 and *p* = 0.224, respectively). However, the thick eyebrow hair count was significantly lower (*p* = 0.043), while the thin hair count was significantly higher (*p* = 0.026) during the follow-up visit than after 10 irradiations. The results are shown in Table 2 and Figure 1.

For the patient with total eyebrow hair loss, eight eyebrow hairs remained at the time of the post-treatment follow-up visit. Of the patients with almost total eyebrow hair loss, two did not experience a change in total eyebrow hair count during the post-treatment follow-up visit, while three experienced a decrease of 1 to 10. Five patients with partial eyebrow hair loss had their total eyebrow hair counts increase by 12 to 40 eyebrow hairs, and for five patients, it decreased by 4 to 31 eyebrow hairs.

All subjects tolerated the treatment well. During the irradiation, patients reported pleasant warmth. The patients did not report any ophthalmologic complaints or headaches during or after LED irradiations.

## 4. Discussion

Eyebrows are made up of short, thick, terminal hairs that serve many functions, including protecting the eyes, improving aesthetic appearance, and assisting with nonverbal communication [8,20,22]. Complete or partial eyebrow loss is observed in the majority of patients with FFA. Clinically, eyebrow involvement appears to be non-scarring and non-inflammatory; however, histopathological examinations revealed the presence of an active perifollicular lymphocytic infiltrate as well as fibrous tracts at the isthmus and supraisthmic areas, which implies a scarring form of eyebrow loss [23]. Interestingly, in another study, Katoulis et al. [24] demonstrated the preservation of sebaceous glands in 38% of eyebrow biopsies in FFA. Considering that the destruction of the sebaceous gland is thought to be the earliest histological sign of scarring alopecia, its preservation may suggest a possibility for eyebrow regrowth in some cases of FFA [24]. A number of treatments have been proposed to control eyebrow loss in FFA, including steroid injections, oral minoxidil, surgical management, topical treatment, and aesthetic camouflage; however, the results are often variable and contentious [9,22,25,26,27].

In this study, we evaluated the efficacy of LEDs on eyebrow involvement in FFA. To the best of our knowledge, this is the first study focusing on the treatment of eyebrow loss in FFA using LED therapy. The 6-month follow-up period permitted the assessment the of long-lasting effects of this therapy. Moreover, this study included 16 patients with FFA, which makes this the largest group studied to evaluate treatment options for eyebrow involvement in FFA. All these aspects contribute to the novelty of our study. After a treatment course consisting of 10 LED irradiations, a significant increase in the total number of hairs was observed. Trichoscopic examination revealed an increased number of thick and mid-thick hairs, which led to clinical improvement. This could be explained as the hair cycle being modulated by the LED therapy [18]. It is presumed that LLLT irradiation prolongs the duration of the anagen phase, stimulates anagen reentry in telogen hair, prevents premature catagen, and increases rates of proliferation in active anagen hair [12,28]. Such a modulation of the hair cycle leads to an increased hair diameter and density [12], which is in accordance with our results. Compared with the scalp, the eyebrow hair cycle is shorter, with the anagen phase lasting 4 weeks and the telogen phase lasting up to 3 months. Moreover, unlike the scalp, 90% of eyebrow hair follicles are in the telogen phase while only 10% are in the anagen phase [22]. It is likely that the significant reduction in the number of thick hairs we observed during the post-treatment follow-up visit, might result from the return of eyebrow hair follicles to their normal cycle. However, contrary to expectation, the total number of eyebrow hairs did not change significantly from the number of hairs at the end of the LED therapy. Moreover, the total number of hairs remained significantly higher than before treatment. This suggests that the effects of LED therapy might not only result from regulation of the hair cycle. Chew et al. [23] reported the presence of active perifollicular lymphocytic infiltrate, perifollicular fibrosis, and focal interface changes in the histopathologic examination of eyebrows affected by FFA, which is similar to findings on the scalp. Currently, it is presumed that the collapse of immune privilege in the bulge region and inflammation-mediated damage to the epithelial stem cells of the hair follicle are responsible for the scarring and irreversible hair loss in FFA [29]. It is suggested that one of the most important mechanisms in immune privilege collapse is a Th-1-mediated inflammatory attack on hair follicles. Together with a failure in the protective mechanism, including decreased expression of locally immunosuppressive agents and a deficiency in peroxisome proliferator-activated receptor gamma (PPAR-y), this leads to permanent destruction of the bulge region of the hair follicle [29,30,31]. It has been suggested that interferon gamma (INF-y) and PPAR-γ-targeted therapy might be a promising strategy in FFA [30]. On a murine model, it was demonstrated that LLLT irradiation exerts an anti-inflammatory effect characterized by a reduction in INF-y levels in the inflammatory infiltrate [32]. In another study, authors found increased PPAR-y expression after LLLT irradiation [33]. Therefore, it cannot be ruled out that LED therapy might modulate those processes and slow down the progression of eyebrow loss in FFA as observed in our patients.

Considering the trichoscopic pattern, only patients who presented whitish areas with an absence of follicular openings within the eyebrow experienced regrowth of single hairs. Clinically, they presented a total or almost total loss of eyebrows. The rest of the patients with partial eyebrow loss experienced clinically and cosmetically acceptable eyebrow regrowth that lasted at least 6 months. Our results suggest that LED therapy enhances eyebrow regrowth in the absence of a fibrosing process in FFA.

In our study, all patients tolerated the therapy well and we did not observe any adverse effects. This is of special interest, since our patients were females aged 60 years or older. In this age group, polymedication and multiple comorbidities could present a challenge. The optimal treatment should be safe and should not interfere with other therapies [2]. With this in mind, LED therapy, as a non-invasive procedure, seems to be a promising therapeutic option for patients with FFA. A healthy appearance of the hair and eyebrows is desirable nowadays, since they determine many aspects of people’s lives [34]. Therefore, the clinical improvement of eyebrow hair observed in our study might enhance not only patients’ physical appearance but also their self-esteem, which in turn may favor social contacts [35].

In our study, we evaluated irradiation with LEDs as a monotherapy. As mentioned above, however, it is likely that LED therapy might also modulate and improve the therapeutic effects of topical or systemic treatments used to treat eyebrow loss in FFA. Nevertheless, further studies are required to check the safety of such a combined therapy. Considering our current results and the results from a previous study, it seems that therapy with LEDs could be a therapeutic option that deals with scalp and eyebrow involvement in FFA in a complex way. Due to the large area of irradiation, LED therapy enables treatment of both eyebrows and the frontal hairline during one treatment session, which might be convenient for patients.

This study has some limitations, including the lack of a control group.

## 5. Conclusions

Considering the progressive nature of eyebrow loss in the course of FFA, there is a need for a safe and long-term therapy for patients. Our study revealed that LED therapy seems to be a novel and promising therapeutic option for eyebrow loss in FFA. LED therapy leads to clinically and cosmetically acceptable improvement. Greater improvement was observed in patients with partial eyebrow hair loss than in patients with almost total eyebrow hair loss. Considering these promising results, further randomized controlled studies using different wavelengths or doses of light are needed to determine the long-term effects and to standardize the optimal treatment parameters of LED therapy.

## Figures and Tables

**Table 1:** Characteristics of the study group (N = 16).

Characteristic, Parameter	Result
Age (years), Min–Max, Median (IQR)	60–74, 65 (62–70)
FFA duration (years), Min–Max, Median (IQR)	3–10, 6 (5–7)
Eyebrow loss duration (years), Min–Max, Median (IQR)	3–10, 6 (5–7)
FFASS, Min–Max, Median (IQR)	4.7–19.3, 12.0 (10.0–15.0)
Permanent eyebrow makeup, n (%)	5 (31.25)
Facial papules, n (%)	7 (43.75)
Reddish dots, n (%)	4 (25.00)
Greyish dots or yellow dots, n (%)	14 (87.50)
Eyebrow regrowth in distinct direction, n (%)	11 (68.75)
Whitish areas with absence of follicular openings, n (%)	6 (37.50)
Comorbidities, n (%)	12 (75.00)
Hypertension, n (%)	8 (50.00)
Hypothyroidism, n (%)	3 (18.75)
Diabetes mellitus, n (%)	1 (6.25)
Obesity, n (%)	1 (6.25)
Hyperlipidemia, n (%)	1 (6.25)
Hiatal hernia, n (%)	1 (6.25)
Depression, n (%)	1 (6.25)
Spinal osteoarthritis, n (%)	1 (6.25)
Knee osteoarthritis, n (%)	1 (6.25)
Balance disorders, n (%)	1 (6.25)
Psoriasis, n (%)	1 (6.25)

IQR—interquartile range (lower quartile–upper quartile).

**Table 2:** Comparison of the total eyebrow hair count and hair counts depending on hair thickness before the treatment, after the treatment, and during the post-treatment follow-up visit (N = 16).

Eyebrow Hair Count	Time	Min-Max, Median (IQR)	*p*		
			Before vs. After	Before vs. Follow-Up	After vs. Follow-Up
Total	Before the treatment	0–369, 132 (29–197)	0.002	0.002	0.623
	After end of the treatment	4–408, 152 (41–253)			
	Follow-up visit	4–404, 176 (38–268)			
Thick	Before the treatment	0–188, 80 (19–107)	0.002	0.033	0.043
	After end of the treatment	0–229, 111 (21–165)			
	Follow-up visit	0–176, 104 (16–160)			
Mid-thick	Before the treatment	0–148, 28 (15–66)	0.044	0.019	0.224
	After end of the treatment	0–157, 34 (22–65)			
	Follow-up visit	0–157, 44 (16–80)			
Thin	Before the treatment	0–38, 6 (1–20)	0.999	0.038	0.026
	After end of the treatment	0–43, 9 (2–16)			
	Follow-up visit	0–83, 16 (2–34)			

IQR—interquartile range (lower quartile–upper quartile).

## Data Availability

The data presented in this study are available from the corresponding author upon request. The data are not publicly available due to privacy restrictions.
